# Variations in the archaeal community and associated methanogenesis in peat profiles of three typical peatland types in China

**DOI:** 10.1186/s40793-023-00503-y

**Published:** 2023-06-06

**Authors:** Xuhui Chen, Dan Xue, Yue Wang, Qing Qiu, Lin Wu, Meng Wang, Jiawen Liu, Huai Chen

**Affiliations:** 1 CAS Key Laboratory of Mountain Ecological Restoration and Bioresource Utilization and Ecological Restoration Biodiversity Conservation Key Laboratory of Sichuan Province, Chengdu Institute of Biology, Chinese Academy of Sciences, No. 9, Section 4, South Renmin Road, Chengdu, 610041 China; 2 Zoige Peatland and Global Change Research Station, Chinese Academy of Sciences, Hongyuan, 624400 China; 3 School of Forestry and Horticulture, Hubei Minzu University, Enshi, 445000 Hubei China; 4 State Environmental Protection Key Laboratory of Wetland Ecology and Vegetation Restoration, Institute for Peat and Mire Research, Northeast Normal University, Changchun, 130024 China; 5 SQE Department, COFCO Coca-Cola Beverages (Sichuan) Company Limited, Chengdu, 610500 China; 6 CAS Center for Excellence in Tibetan Plateau Earth Sciences, Chinese Academy of Sciences (CAS), Beijing, 100101 China; 7 University of Chinese Academy of Sciences, Beijing, 100049 China

**Keywords:** Peatland, Methane, Archaeal community, Peat properties, Methanogenesis

## Abstract

**Background:**

Peatlands contain about 500 Pg of carbon worldwide and play a dual role as both a carbon sink and an important methane (CH_4_) source, thereby potentially influencing climate change. However, systematic studies on peat properties, microorganisms, methanogenesis, and their interrelations in peatlands remain limited, especially in China. Therefore, the present study aims to investigate the physicochemical properties, archaeal community, and predominant methanogenesis pathways in three typical peatlands in China, namely Hani (H), Taishanmiao (T), and Ruokeba (R) peatlands, and quantitively determine their CH_4_ production potentials.

**Results:**

These peatlands exhibited high water content (WC) and total carbon content (TC), as well as low pH values. In addition, R exhibited a lower dissolved organic carbon concentration (DOC), as well as higher total iron content (TFe) and pH values compared to those observed in T. There were also clear differences in the archaeal community between the three peatlands, especially in the deep peat layers. The average relative abundance of the total methanogens ranged from 10 to 12%, of which *Methanosarcinales* and *Methanomicrobiales* were the most abundant in peat samples (8%). In contrast, *Methanobacteriales* were mainly distributed in the upper peat layer (0–40 cm). Besides methanogens, *Marine Benthic Group D/Deep-Sea Hydrothermal Vent Euryarchaeotic Group 1* (*MBG–D/DHVEG–1*), *Nitrosotaleales*, and several other orders of *Bathyarchaeota* also exhibited high relative abundances, especially in T. This finding might be due to the unique geological conditions, suggesting high archaeal diversity in peatlands. In addition, the highest and lowest CH_4_ production potentials were 2.38 and 0.22 μg g^−1^ d^−1^ in H and R, respectively. The distributions of the dominant methanogens were consistent with the respective methanogenesis pathways in the three peatlands. The pH, DOC, and WC were strongly correlated with CH_4_ production potentials. However, no relationship was found between CH_4_ production potential and methanogens, suggesting that CH_4_ production in peatlands may not be controlled by the relative abundance of methanogens.

**Conclusions:**

The results of the present study provide further insights into CH_4_ production in peatlands in China, highlighting the importance of the archaeal community and peat physicochemical properties for studies on methanogenesis in distinct types of peatlands.

**Supplementary Information:**

The online version contains supplementary material available at 10.1186/s40793-023-00503-y.

## Introduction

Climate change has attracted considerable attention from researchers worldwide due to its great impacts on natural ecosystems and human society [1–3], with more and more climate-associated studies carried out [4–6]. Indeed, comprehensive studies on greenhouse gas emissions are of great significance to better understand climate change and accurately predict future trends of global warming [3, 7, 8].

Methane (CH_4_) is the second most abundant greenhouse gas after CO_2_, contributing to the radiative force increase in the lower atmosphere by about 20% [8, 9]. The global warming potential (GWP) of CH_4_ is about 28 times higher than that of CO_2_ over a 100-year time horizon [3, 10, 11]. In addition, the CH_4_ concentration in the atmosphere has increased by at least 2.5 times since the first industrial revolution in 1750 and is predicted to reach 2000 ppb by 2030 [8, 12].

Peatland is an important carbon sink worldwide, stocking about 500 Pg of carbon, even though it covers 3% of the land surface [13, 14]. However, peatland can also be a potential source of CH_4_, with an estimated annual CH_4_ flux of about 3.6 mg m^−2^ [15], emitting approximately 126 Tg CH_4_ per year, which contributes to at least 50 and 14–27% of CH_4_ emitted from wetlands and global, respectively [16–18]. Therefore, investigating peatland-climate links is of great significance for predicting future climate change, as well as for the fate and evolution of peatlands.

Peatlands can be generally classified as minerotrophic fen, ombrotrophic bog, and other intermediate types depending on the climatic, hydrological, and topographical conditions [13, 19, 20]. Indeed, there are significant differences between minerotrophic fens and ombrotrophic bogs. For example, the pH of minerotrophic fens is higher than that of ombrotrophic bogs due to the mineral enrichment from groundwater [21, 22], while atmospheric precipitation dominates in ombrotrophic bogs, resulting in low pH values [15, 23]. Subsequently, the water recharge and pH conditions can further influence vegetation colonization, as grass-sedge and *Sphagnum* dominates in fens and bogs, respectively, reflecting their nutrient conditions [13, 19, 24].

According to Kolka et al. [15] about 90% of peatlands are located in boreal regions, making these regions research hotspots. However, peatlands in China receive considerably less attention from researchers, although they cover an area of more than 10^5^ km^2^ [19]. Peatlands in China are mainly distributed in the Eastern Tibetan Plateau, Yungui Plateau, Coastal Plain of Fujian-Guangdong, Yangtze Plain, and the Northeast Region [25], covering various peatland types [25–27]. However, systematic studies on CH_4_ production processes in these regions are limited. Moreover, it is necessary to determine the ecological attribute of peatlands to better understand their potential contributions to the carbon cycle and future climate change in China.

CH_4_ production in peatlands is quite different from CH_4_ emission, as most of the CH_4_ produced in peatlands is consumed by microbes, then the remaining amount can eventually be emitted to the atmosphere [28–30]. Therefore, CH_4_ production is the primary and direct factor of CH_4_ emission in peatlands. In nature, CH_4_ is produced by methanogens from seven orders of *Euryarchaeota*, namely *Methanobacteriales*, *Methanococcales*, *Methanopyrales*, *Methanosarcinales*, *Methanomicrobiales*, *Methanocellales*, and *Methanomassiliicoccales* [31–33]. However, new archaea that can also produce CH_4_ have recently been identified, such as *Methanomethyliales* and *Methanofastidiosales*, as well as several species belonging to *Bathyarchaeota* [32, 33]. Although there are various methanogens in taxonomy, the metabolic pathways of CH_4_ production can be generally classified as hydrogenotrophic, acetotrophic, methylotrophic, methoxydotrophic, and alkylotrophic methanogenesis, according to the substrates used for metabolisms [34–37]. Peatlands provide anoxic environments in which methanogens are highly adapted due to the permanent water-saturated conditions [13, 34]. Therefore, it is important to explore the ecological and biological controls associated with CH_4_ production in peatlands.

Several factors affect CH_4_ production in peatlands, including temperature [38–40], hydrological characteristics [41, 42], and electron acceptor availability [43–45]. However, these factors cannot comprehensively explain CH_4_ production in peatlands [44, 46–48]. Therefore, further comprehensive studies are required to reveal the mechanisms controlling CH_4_ production in peatlands. In addition, further studies on the investigation of the main factors controlling CH_4_ production in peatlands at the microbial scale are required to determine the relationships between biological and ecological functions, as well as the corresponding processes in peatlands.

The present study aims to explore the potential impacts of methanogens, as well as other archaea, and basic physicochemical properties on CH_4_ production in three typical peatlands in China. The differences in the archaeal community and CH_4_ production processes were determined in this study to reveal the characteristics of the archaeal community associated with CH_4_ production in the study areas. Here, we hypothesis that the archaeal communities are substantially distinct among three typical peatland types, and the differences of methanogenesis among them can be explained by the physicochemical properties and the archaeal communities.

## Materials and methods

### Study area

**Fig. 1:**
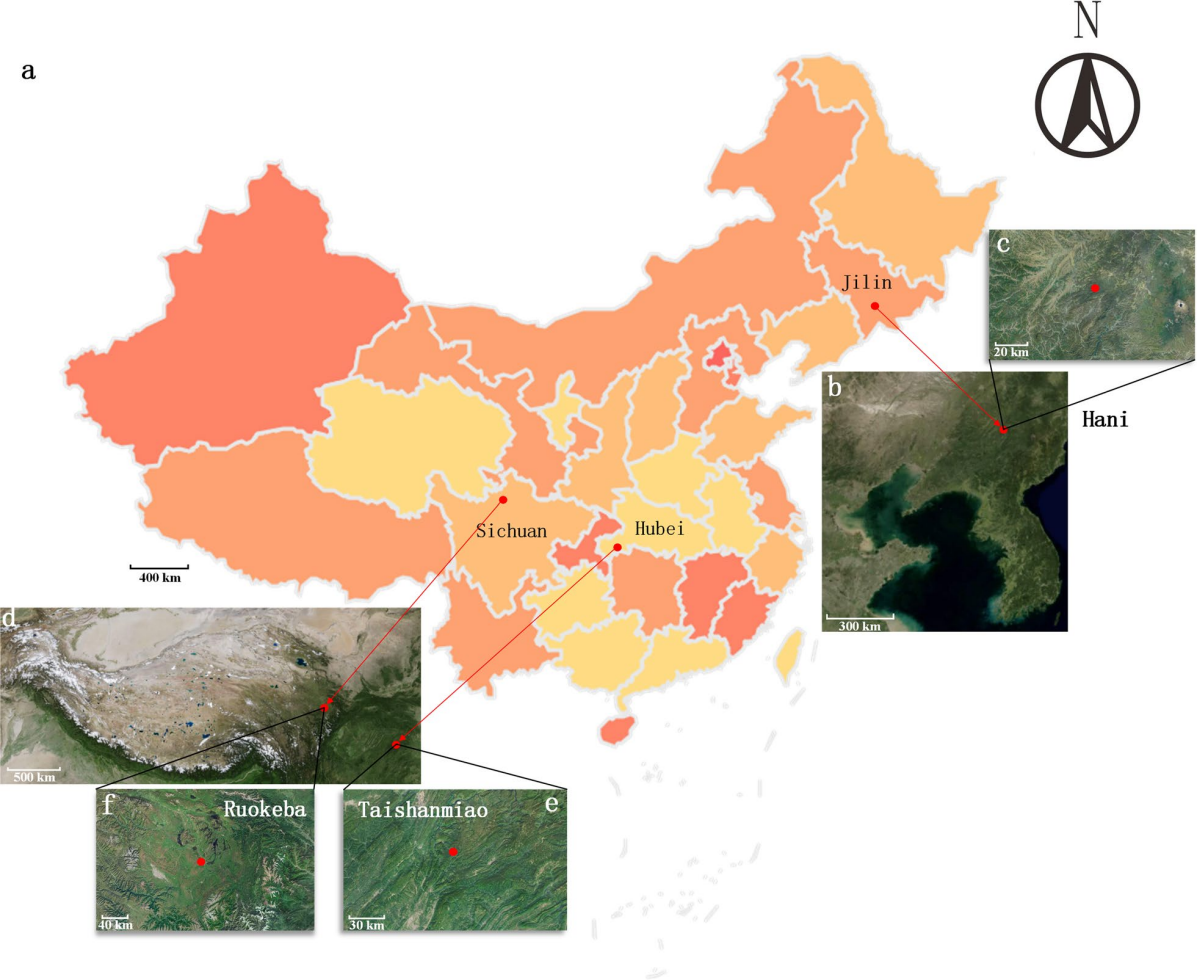
Geographic locations of the sampling sites in China. **a** Administrative map of China; **b** Satellite images of Bohai Bay and the Eastern Liaoning Peninsula; **c** Satellite image of Hani peatland; **d** Satellite image of Qinghai–Tibet Plateau; **e** Satellite image of Taishanmiao peatland; **f** Satellite image of Ruokeba peatland. Red points indicate the three sampling sites. The colors on the map indicate the provincial boundaries

In this study, we selected three typical peatlands in China (Fig. 1a), namely Hani (H), Taishanmiao (T), and Ruokeba (R) peatlands. H is located in Tonghua City, Jilin Province, in the western part of Changbai Mountain (Fig. 1b and c). The selected peatland covers an area of about 18 km^2^ at 926 m asl [49]. The basement is constituted by volcanic eruption-derived basalt during the Cenozoic Era, and the thickness of the peat layer can reach several meters [50, 51]. Sedge-*Sphagnum* is the dominant vegetation species in this study area [27, 50–52] H can be classified as a poor fen. T is located in Enshi Tujia and Miao Autonomous Prefecture, Hubei Province at 1826 m asl (Fig. 1d and e). This peatland is comparatively recent and unevenly distributed, with thickness of peat hardly exceeding 1 m. *Sphagnum* is the absolute dominant plant [27].Underground karst topography may have impact on this peatland [26, 27]. T can be classified as an ombrotrophic bog.R is located in Ngawa Tibetan and Qiang Autonomous Prefecture, Sichuan Province, in the southern part of the Zoige Basin at 3467 m asl (Fig. 1d and f). Precipitation events occur mainly in summer. This peatland receives few disturbances from human activities, with a comparatively long development period. The thickness of the peat layer can reach several meters [53]. The vegetation in R is dominated by sedge and grass.R is classified as a minerotrophic fen. More detailed information about the three peatlands can be found in Additional file 1: Table S1.

### Collection and preparation of peat samples

Peat soil samples were collected from H and T during the growing season in 2020, and R in 2019. Indeed, 3 sampling sites on flat terrains, with similar vegetation composition and plant growing conditions, were randomly selected in this study. The distance between sampling sites was at least 20 m. Specifically, a 5 × 5 m quadrat was randomly placed at each site, from which five peat samples were collected following a diagonal sampling pattern, then mixed to obtain a composite sample from each sampling site. To obtain the complete characteristics of archaeal community along the peat profile across surface, transitional, and deep layers, we collected the 1 m-depth peat from three peatlands. Specifically, vegetation above the peat surface was first removed, then a stainless-steel soil drill was used to collect samples from five consecutive pear layers, namely 0–20, 20–40, 40–60, 60–80, and 80–100 cm. The peat samples were placed in plastic boxes and kept refrigerated, then immediately transferred to the laboratory.

In the laboratory, vegetation roots and debris were removed. A random part of each sample was placed in an ultra-low temperature freezer at − 80 °C for microbiological analysis, while the remaining part of the peat sample was dried and sieved using a 2 mm plastic sieve. In addition, a part of each sieved soil sample was sieved through a 0.15 mm plastic sieve for physicochemical analyses.

### Determination of peat physicochemical properties

A portable pH meter (OxyScan 300, UMS GmbH & Co. KG., Germany) was used to determine the pH values of the peat samples. Whereas the gravimetric method was used to determine the peat water content (WC). Dissolved organic carbon (DOC) was determined by a total organic carbon (TOC) analyzer (vario TOC, Elementar Analysensysteme GmbH, Germany) after extraction with ultrapure water [54]. The total iron contents (TFe) in peat samples were determined using the ultraviolet spectrophotometry method [55]. Total carbon content (TC) and total nitrogen content (TN) were determined by the elementary analyzer (Vario Cube, Elementar Analysensysteme GmbH, Germany). The total phosphorus (TP) contents were determined using an elementary analyzer following extraction using the modified sulphuric acid-perchloric acid digestion method [56, 57].

### Incubation experiment

An incubation experiment was conducted in this study to quantify the CH_4_ production potential of the three peatlands. All layers of peat in each peatland were first well-mixed to form a 1 m-depth integral sample. Peat samples (20 g) were placed into brown incubation flasks with 40 mL ultrapure water [58]. The headspace was purged with nitrogen through a gas displacement device. In addition, a pre-incubation period of 7 days at 15 °C and under 150 rpm in the incubator shaker was considered in this study to ensure proper microbe activation and peat sample mixing [59]. The incubation experiment was conducted over 104 days and at 25 °C to obtain the potential of methane production. The gas in the headspace was collected weekly, then stored in vacuum tubes and analyzed using a gas chromatograph (Clarus 500, PerkinElmer Co., USA). The headspace in the flask was replaced by N_2_ after gas sampling [58, 60].

The methanogenesis pathways were determined in the present study. 5 g of mixed peat samples were placed into 100 mL brown incubation flasks with 30 mL ultrapure water or solutions (M:V of 1:6) [58, 61]. Indeed, one control and three treatment groups were considered in the experiment. 30 mL ultrapure water was added to the control group (CK; 5 g peat + 30 mL water), while the three treatment groups consisted of methanol (Me; 5 g peat + 30 mL methanol), acetate (Ac; 5 g peat + 30 mL acetate), and sodium bicarbonate (CO_2_; 5 g peat + 30 mL sodium bicarbonate), with final solution concentrations of 0.02 mol L^−1^ [62, 63]. Each group consisted of three parallel samples, which were incubated at 25 °C. Before incubation, the pH values of all samples were adjusted to the in-situ pH values [64]. For the CO_2_ group, 10 mL of H_2_ was injected following the replacement of headspace gas. The gas in the headspace was collected on 3, 7, 14, and 21 days of incubation and analyzed using a gas chromatograph.

### Metagenome sequencing

#### DNA extraction

The PowerSoil DNA Isolation Kit (MO BIO Laboratories Inc., the Netherlands) was used in this study to extract DNA from peat samples following the manufacturer’s instructions. The extracted DNA was stored at − 80 °C until further processing. The quantity and quality of the extracted DNA were determined by a NanoDrop 1000 (Thermo Fisher Scientific Co., USA) and 1.2% agarose gel electrophoresis, respectively.

#### Paired-end sequencing with illumina MiSeq

The TruSeq Nano DNA LT Library Prep Kit was used to prepare 16S rRNA gene sequencing libraries, following the Illumina official protocol (Illumina Co., USA). The 16S rRNA gene primer pair, 524F(5′-TGYCAGCCGCCGCGGTAA-3′)/958R(5′-YCCGGCGTTGAVTCCAATT-3′), was used to amplify the V4V5 regions. The paired-end libraries were then selected and purified through 2% agarose gel electrophoresis. Before sequencing, the quality of the libraries was tested using a High Sensitivity DNA Kit (Agilent Technologies Co., USA). The libraries had only one single peak without adaptors. Next, a Promega QuantiFluor (Promega Co., USA) with Quant-iT PicoGreen dsDNA Assay Kit (Molecular Probes Co., USA) was used to quantify the selected libraries with concentrations ≥ 2 nmol L^−1^. All samples were first pooled in equimolar concentrations and denatured into single strands by NaOH solution, then sequenced on the Illumina MiSeq platform using MiSeq Reagent Kit V3 (600 cycles, Illumina Co., USA).

#### Processing of sequencing data

The sequence data were processed using QIIME 2 2019.4 [65], with slight modifications to the official tutorials (https://docs.qiime2.org/2019.4/tutorials/). Raw sequence data were first demultiplexed and filtered using the demux plugin then the primers were cut using the cutadapt plugin [66]. All amplicon sequence variants (ASVs) were aligned with mafft via the q2-alignment plugin [67]. The q2-diversity plugin was used to estimate *α*-diversity and *β*-diversity metrics of 900 sequences per sample with Bray–Curtis dissimilarity following sampling without replacement [68, 69]. Taxonomy was assigned to ASVs using the q2-feature-classifier plugin with 99% similarity in operational taxonomy units (OTUs) of sequence against the Greengenes 13_8 database [70, 71].

### Data processing and analysis

The produced CH_4_ amounts were estimated in this study using the following formula [58]:

1 $${\text{P}}\; = \;{\text{c}} \cdot \frac{{\text{V}}}{{\text{W}}} \cdot \frac{{{\text{MW}}}}{{{\text{MV}}}} \cdot \frac{{{\text{T}}_{0} }}{{\text{T}}}$$

where P (μg g^−1^) denotes the CH_4_ concentration in the headspace; c (ppmv) denotes the CH_4_ concentration determined by gas chromatograph; V (L) denotes the volume of headspace; W (g) denotes the dry weight of the peat sample; MW (16 g mol^−1^) denotes the relative molecular mass of CH_4_; MV (22.4 L mol^−1^) denotes the molar volume of gas under standard conditions; T_0_ (273.15 K) is the Kelvin temperature under standard conditions; T (K) is the incubation temperature.

The weekly CH_4_ production rates were further summed to draw the accumulation–incubation time curve of CH_4_. The *grofit* R package was used to estimate the CH_4_ production potential, representing the highest CH_4_ rate [72]. For the methanogenesis pathway experiment, the accumulation–incubation time of CH_4_ was fitted using linear regression, showing an average determination coefficient (*R*^2^) of 0.94. The average CH_4_ production rate of each control group was used to normalize the values of corresponding samples in treatment groups to obtain relative methanogenesis values. This was only a qualitative method to determine the dominant methanogenesis pathways in the three considered peatlands.

The *β*-diversity of archaea was determined using a nonmetric multidimensional scaling (NMDS) conducted by the Bray–Curtis dissimilarity matrix based on the OTU of the archaeal community. Differences in archaea abundance between different areas were shown in this study using a heatmap, following data normalization using the *Z*-score method. In addition, clustering trees were performed in this study using the unweighted pair-group method with arithmetic means (UPGMA) based on the Bray–Curtis dissimilarity matrix of species and areas to reveal the similarity in the peat samples and the distributions of the archaeal community. Pearson correlation analysis was performed in this study using the *corrplot* package [73] in R [74].

Maps and satellite images were captured from DataV.GeoAtlas (Alibaba Cloud Computing Co., China). Statistical analyses, tables, and figures were all performed in Excel 2019 (Microsoft Co., USA), R v3.6.3 [74], and Visio 2019 (Microsoft Co., USA).

## Results

### Physicochemical properties of the three peatlands

The WC in H, T, and R was generally high, ranging from 300 to 700% in most peat soil samples (Table 1). TC showed an increasing trend with soil depth in both H and R, ranging from 20 to 40%. In contrast, a decreasing trend in the TC values was observed in T, varying from 30 to 9.66% (Table 1). The change in the TN content was similar to that of TC in the three peatlands, ranging from 0.61 to 2.20% (Table 1). The TP contents showed a decreasing trend with soil depth, ranging from 0.40 to 1.26%. However, this variation was not observed in H (Table 1). The carbon/nitrogen (C/N) values of R showed a significant increase with soil depth (*p* < 0.05), varying from 15 to 18.61. In contrast, no significant differences in the C/N values were observed between the soil layers of the remaining peatlands, showing values of about 17 across the soil profiles (Table 1). In T, the DOC concentrations in the deep soil layers (40–100 cm) were significantly lower than those in the surface layers (0–20 cm, *p* < 0.05), ranging from 1.13 to 3.04 mg g^−1^, while no significant differences in the DOC concentrations were observed between the soil layers in H and R (Table 1). Soils in R exhibited higher pH (mean 5.92) and TFe (mean 2.62 mg Fe g^−1^) than those observed in the other two peatlands, regardless of depth (*p* < 0.05, Table 1). In addition, the obtained results showed significantly higher TFe contents in the upper soil layer than those in the deeper soil layers in H and T (*p* < 0.05). Whereas in T, significantly lower TFe contents in the upper soil layers than those in the deeper soil layers were observed (*p* < 0.05, Table 1).

**Table 1:** Peat physicochemical properties in different peat layers in the three peatlands

Location	Depth (cm)	WC (%)	TC (%)	TN (%)	TP (mg g^−1^)	C/N	DOC (mg g^−1^)	pH	TFe (mg Fe g^−1^)
H	0–20	643.32 ± 12.87^ab^	28.70 ± 0.77^c^	1.67 ± 0.04^b^	0.61 ± 0.07^a^	17.18 ± 0.80^a^	1.84 ± 0.32^a^	5.71 ± 0.38^a^	2.66 ± 0.74^a^
H	20–40	539.65 ± 35.38^b^	30.67 ± 0.1^bc^	1.89 ± 0.10^ab^	0.67 ± 0.06^a^	16.33 ± 0.85^a^	1.33 ± 0.27^a^	4.97 ± 0.10^b^	1.34*
H	40–60	644.64 ± 35.38^ab^	34.00 ± 0.93^ab^	2.10 ± 0.08^a^	0.60 ± 0.07^a^	16.20 ± 0.26^a^	1.69 ± 0.15^a^	5.12 ± 0.06^b^	1.32 ± 0.13^b^
H	60–80	631.58 ± 58.60^ab^	34.22 ± 1.60^ab^	2.00 ± 0.12^ab^	0.56 ± 0.03^a^	17.17 ± 0.54^a^	1.59 ± 0.07^a^	5.10 ± 0.08^b^	1.35 ± 0.18^b^
H	80–100	705.02 ± 41.32^a^	36.54 ± 0.59^a^	2.08 ± 0.09^a^	0.55 ± 0.08^a^	17.59 ± 0.80^a^	1.88 ± 0.42^a^	4.91 ± 0.09^b^	1.50 ± 0.15^b^
H	Mean	632.87 ± 66.30^A^	32.83 ± 3.19^A^	1.95 ± 0.21^A^	0.60 ± 0.07^B^	16.89 ± 1.15^A^	1.67 ± 0.31^A^	5.16 ± 0.33^B^	1.60 ± 0.56^B^
T	0–20	937.34 ± 191.97^a^	30.90 ± 4.21^a^	1.65 ± 0.20^a^	0.91 ± 0.05^a^	18.69 ± 0.26^a^	3.04 ± 0.49^a^	5.09 ± 0.23^a^	2.10 ± 0.75^a^
T	20–40	713.11 ± 361.53^ab^	29.35 ± 6.23^a^	1.64 ± 0.29^a^	1.00 ± 0.05^a^	17.69 ± 0.87^a^	2.34 ± 1.18^ab^	4.77 ± 0.31^a^	0.73 ± 0.02^ab^
T	40–60	345.84 ± 168.14^bc^	22.50 ± 4.02^a^	1.34 ± 0.28^a^	0.96 ± 0.04^a^	16.96 ± 0.55^a^	1.25 ± 0.47^b^	5.01 ± 0.14^a^	0.57 ± 0.13^b^
T	60–80	205.78 ± 102.93^bc^	17.28 ± 6.63^a^	0.96 ± 0.33^a^	0.61 ± 0.14^ab^	17.31 ± 0.99^a^	1.13 ± 0.31^b^	5.04 ± 0.22^a^	0.39 ± 0.21^b^
T	80–100	121.76 ± 124.82^c^	9.66 ± 6.50^a^	0.61 ± 0.33^a^	0.40 ± 0.10^b^	13.61 ± 2.36^a^	1.28 ± 0.12^b^	5.09 ± 0.34^a^	0.52 ± 0.17^b^
T	Mean	464.80 ± 368.10^B^	21.94 ± 11.58^B^	1.24 ± 0.60^B^	0.78 ± 0.27^A^	16.85 ± 2.54^A^	1.81 ± 0.94^A^	5.00 ± 0.25^B^	0.87 ± 0.74^C^
R	0–20	337.12 ± 16.05^a^	28.33 ± 2.37^b^	1.88 ± 0.11^ab^	1.26 ± 0.05^a^	14.99 ± 0.40^b^	0.89 ± 0.07^a^	5.83 ± 0.35^ab^	3.14 ± 0.91^ab^
R	20–40	341.34 ± 129.62^a^	21.13 ± 2.20^b^	1.42 ± 0.10^c^	0.94 ± 0.19^b^	14.80 ± 0.52^b^	0.96 ± 0.27^a^	5.57 ± 0.13^b^	3.31 ± 0.96^a^
R	40–60	304.94 ± 88.69^a^	27.65 ± 0.63^b^	1.64 ± 0.04^bc^	0.71 ± 0.03^bc^	16.93 ± 0.74^ab^	1.01 ± 0.30^a^	6.04 ± 0.05^ab^	1.84 ± 0.06^b^
R	60–80	379.24 ± 37.52^a^	36.19 ± 0.94^a^	1.96 ± 0.07^ab^	0.60 ± 0.05^c^	18.50 ± 0.64^a^	1.27 ± 0.04^a^	6.09 ± 0.17^a^	2.29 ± 0.21^ab^
R	80–100	467.17 ± 33.50^a^	40.91 ± 0.83^a^	2.20 ± 0.03^a^	0.62 ± 0.04^c^	18.61 ± 0.16^a^	1.23 ± 0.04^a^	6.06 ± 0.11^ab^	2.52 ± 0.30^ab^
R	Mean	366.00 ± 85.11^B^	30.84 ± 7.55^A^	1.82 ± 0.30^A^	0.82 ± 0.27^A^	16.76 ± 1.87^A^	1.07 ± 0.22^B^	5.92 ± 0.26^A^	2.62 ± 0.76^A^

Data were presented as mean ± 1 SD (*n* = 3). Different lowercase letters indicate significant differences in each parameter between peat layers in each peatland at the *p* < 0.05 level, while the uppercase letters indicate significant differences in the average value of each parameter between the three peatlands at the *p* < 0.05 level, regardless of depth, determined using the Tukey’s HSD test, with *p* < 0.05. WC, TC, TN, TP, DOC, and TFe denote peat water content, total carbon content, total nitrogen content, total phosphorus content, dissolved organic carbon, and total Fe content, respectively. The C/N values were calculated using the mass ratio (TC/TN). H, T, and R denote Hani, Taishanmiao, and Ruokeba peatlands, respectively

*This value was not considered in the ANOVA test due to sample replicate loss. Only a single value is presented

### Archaeal community characteristics and compositions in the three peatlands

**Fig. 2:**
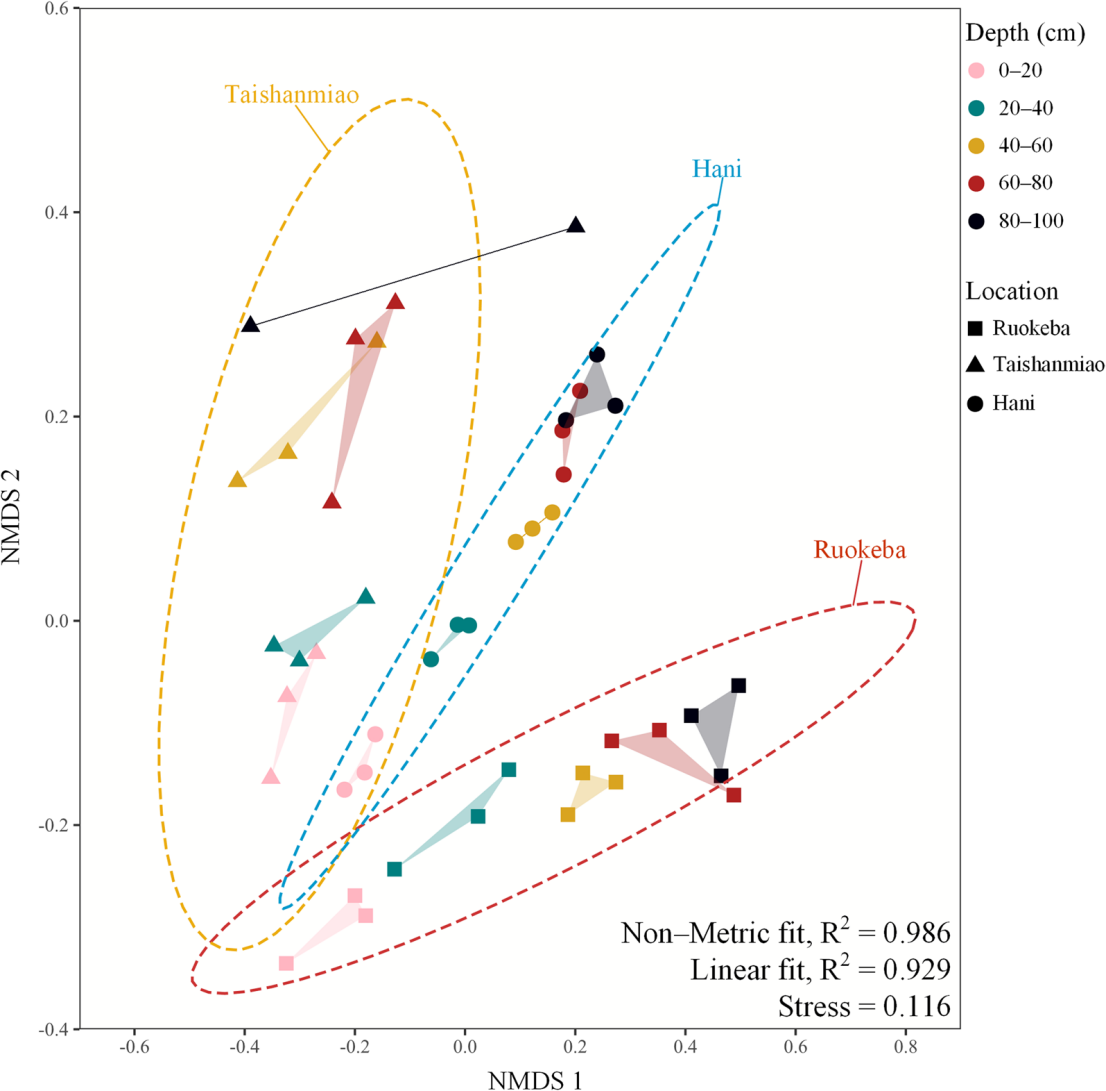
*β*-diversity of the archaeal community in the peat profiles in the three peatlands. It was determined by nonmetric multidimensional scaling (NMDS). Different color points represent different peat depths. Peat samples from Hani, Taishanmiao, and Ruokeba are indicated by circles, triangles, and squares, respectively. The *R*^2^ values were calculated based on the regression between ordination distance and observed original distance using the Shepard plot. Dashed ellipses indicate confidence intervals of samples from each peatland determined using the *t*-test (*α* = 0.05)

To better investigate the *β*-diversity of the archaeal community in the three peatlands, we established a nonmetric multidimensional scaling (NMDS) ordination (Fig. 2). This method intuitively exhibited a comparable archaeal community structure in the three peatlands in which the archaeal community became more distant with soil depth, showing a changing trend with peat depth. The projections of the archaeal community in T and R were set on the two opposite sides, while that in H was in the middle (Fig. 2).

**Fig. 3:**
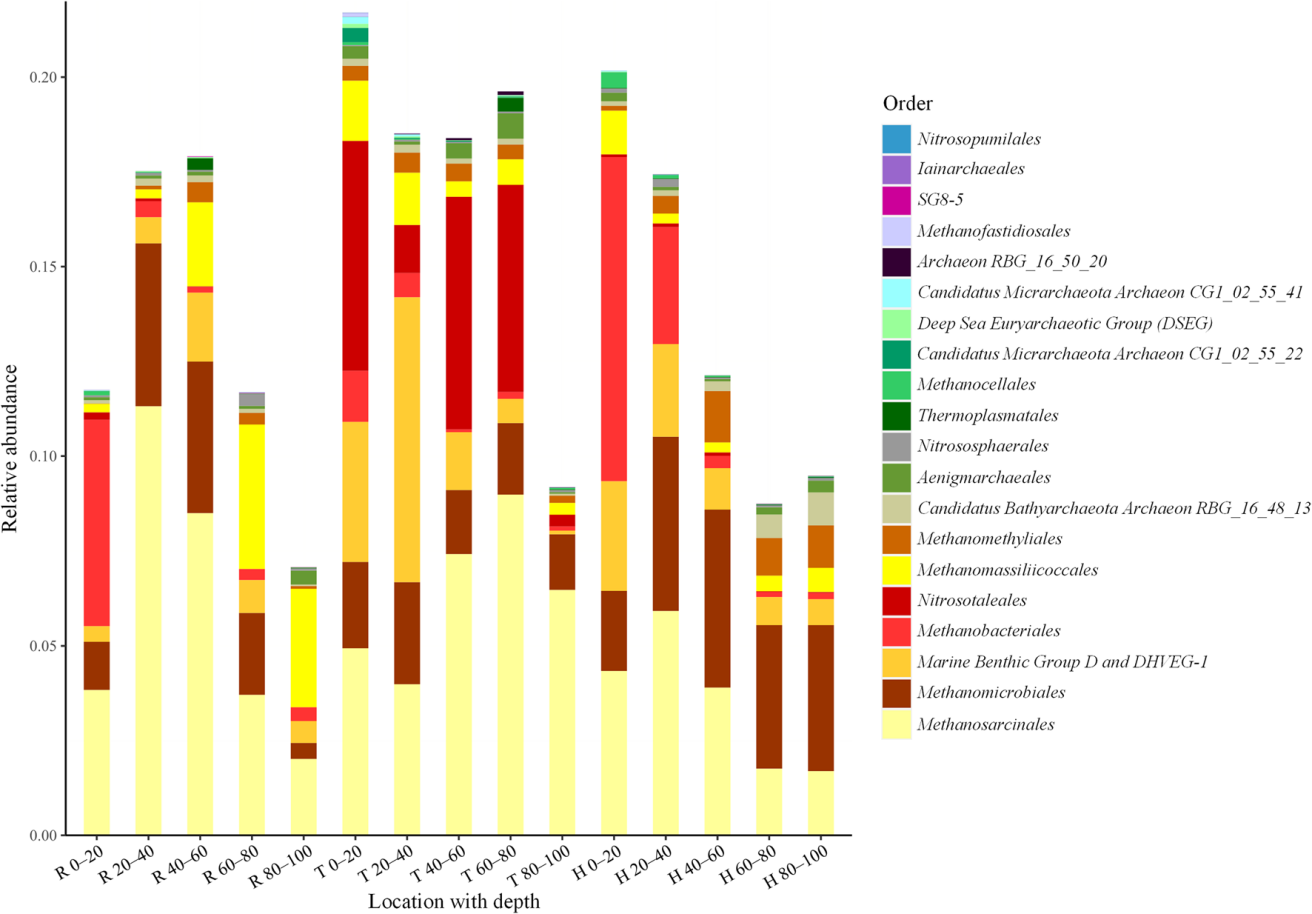
Relative abundances of the top 20 orders of archaea in the three peatlands. Different colors indicate different orders of archaea. The specific locations and peat layers (cm) are reported on the *x*-axis. R, T, and H denote Ruokeba, Taishanmiao, and Hani, respectively

To quantitively assess the archaeal community composition, the top 20 orders of archaea were selected in this study based on their relative abundances (Fig. 3). In general, the dominant archaea were observed mainly in the upper layer of peat soil. There were seven orders of methanogens, with a total relative abundance range of 10–12% in each peatland. Indeed, five orders of these methanogens were included in the top seven orders in the archaeal community. Unlike *Methanobacteriales* that showed a high relative abundance in the 0–40 cm peat layer, *Methanosarcinales* and *Methanomicrobiales* were abundant in all peat samples, while *Methanomassiliicoccales* exhibited a stable distribution across the peat profiles of the three peatlands, with relatively higher relative abundance in the 40–100 cm peat layer of R. A similar distribution was observed for *Methanomethyliales*. The relative abundances of *Methanocellales* and *Methanofastidiosales* were low in all peat samples (< 1%). Besides methanogens, other archaeal communities might exhibit high relative abundances, especially in T, where the relative abundances of the *Marine Benthic Group D/deep-Sea Hydrothermal Vent Euryarchaeotic Group 1* (*MBG–D/DHVEG–1*) and *Nitrosotaleales* reached an average value of 3.3%.

**Fig. 4:**
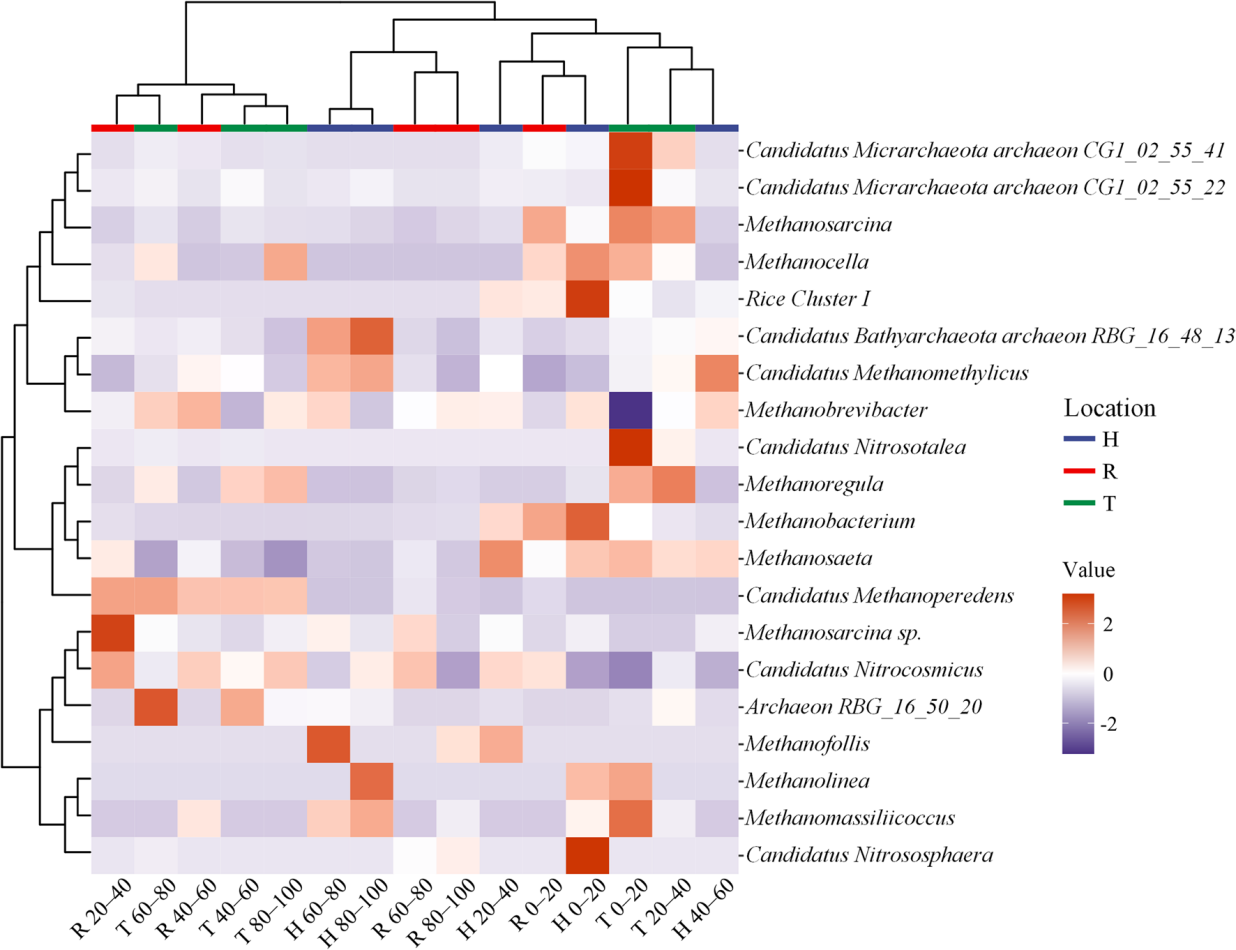
Heatmap of the abundances of the top 20 genera of archaea in the three peatlands. All data were normalized to obtain differentiation values. Different colors show the differences in the archaeal abundances in different peat samples. Red and purple colors represent relatively high and low abundances of the archaeal community, respectively. Bars with different colors at the top of the heatmap indicate different locations of peat samples. Specific locations and peat layers (cm) are reported on the *x*-axis. R, T, and H denote Ruokeba, Taishanmiao, and Hani, respectively

The differences in archaeal distribution between the three peatlands are shown in Fig. 4. Clustering results showed similar compositions of the dominant archaeal communities in the deep peat layer (60–100 cm) of H and R. For example, *Candidatus Bathyarchaeota Archaeon RBG_16_48_13*, *Candidatus Methanomethylicus*, *Methanofollis*, *Methanolinea*, and *Methanomassiliicocus* were more abundant in the deep peat layer of H. On the other hand, similar archaeal community compositions were observed in the upper peat layer (0–40 cm), where *Methanosarcina*, *Methanocella*, *Methanosaeta*, and others were the most abundant species. Furthermore, similar distribution patterns of the archaeal compositions between the 40–100 cm and 20–60 cm peat layers in T and R, respectively, were observed.

### Methane production potentials, dominant pathways, and influencing factors

**Fig. 5:**
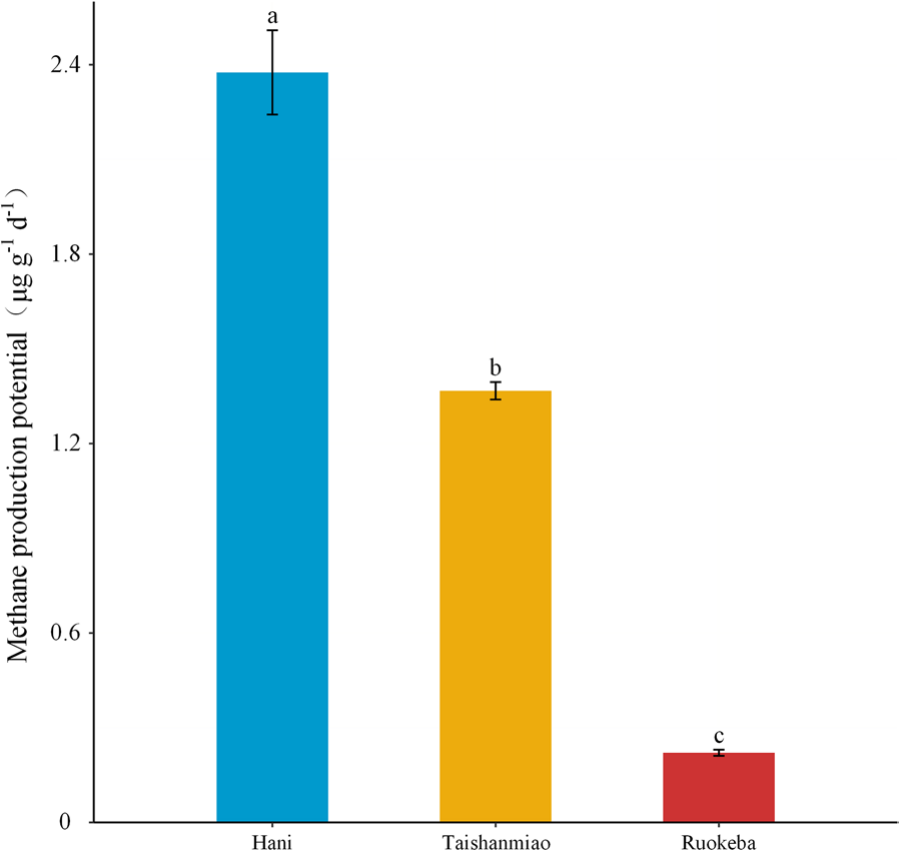
Methane production potentials in the three study peatlands. Different letters indicate significant differences in methane production potentials between peatlands (*p* < 0.05)

The CH_4_ production potentials showed significant variations between the three peatlands. H exhibited a greater CH_4_ production potential than R and T (*p* < 0.05), showing CH_4_ production potential values of 2.38, 0.22, and 1.37 μg^−1^ g^−1^ d^−1^, respectively (Fig. 5).

**Fig. 6:**
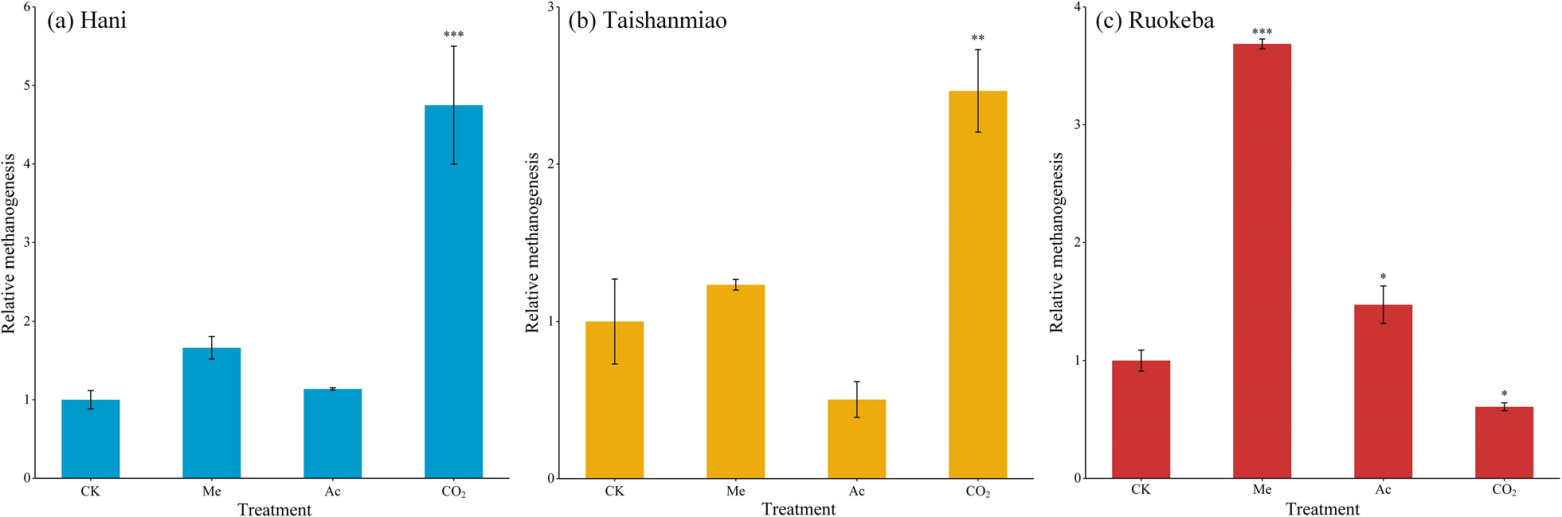
Relative methanogenesis in Hani (**a**), Taishanmiao (**b**), and Ruokeba (**c**) peatlands under different substrate additions. *, **, and *** indicate significant differences in relative methanogenesis between treatments and control groups in each peatland at *p* < 0.05, *p* < 0.01, and *p* < 0.001, respectively. Multiple comparisons were performed using Dunnett’s method between control and treatment groups. CK, Me, Ac, and CO_2_ represent control, methanol-addition, acetate-addition, and sodium bicarbonate/H_2_-addition groups, respectively

According to the relative methanogenesis, determined by the addition of substrates, hydrogenotrophic methanogenesis was the dominant pathway in H, showing a higher CH_4_ production rate than the control group by over 3.7 times (*p* < 0.001, Fig. 6a), while no significant differences in the CH_4_ production rates between the remaining and control groups. A similar result was observed in T, showing greater methanogenesis in the CO_2_ group by about 2.5 times than that in the control group (*p* < 0.01, Fig. 6b). Unlike H_2_/CO_2_ addition, methanol and acetate additions promoted CH_4_ production in R, of which methanogenesis with methanol was significantly more active (*p* < 0.001, Fig. 6c).

Several correlated indices with CH_4_ production potential were filtered in this study. Indeed, WC, pH, and DOC were the main factors associated with CH_4_ production potential (Table 2). The analysis of covariance showed a strong interaction between peat pH and the study areas (*p* = 0.005, Additional file 1: Tables S2 and S3). In terms of methanogens, a significant negative correlation was found between *Methanomassiliicoccales* and CH_4_ production potential (*R* =  − 0.716, *p* < 0.05, Table 2 and Additional file 1: Table S2).

**Table 2:** Correlation coefficients between methane production potential (MPP), environmental factors, and relative abundances of methanogens

	WC	pH	DOC	*Methanomassiliicoccales*
MPP	0.759*	− 0.811**	0.690*	− 0.716*

* and ** indicate significant correlations at *p* < 0.05 and *p* < 0.01, respectively

### Correlation analysis between peat physicochemical properties, archaeal community diversity indices, and archaeal abundances

**Fig. 7:**
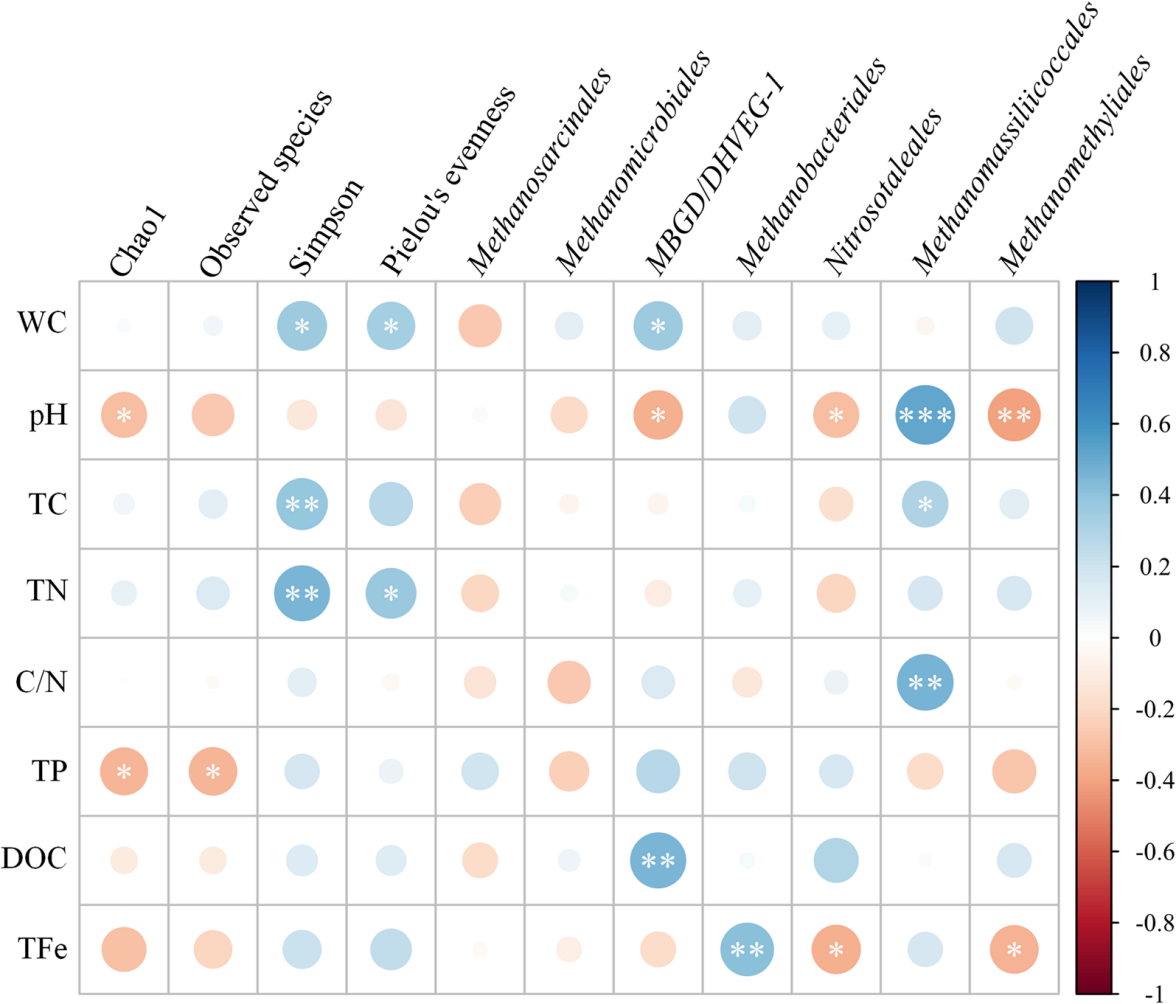
Correlations between physicochemical properties, archaeal community diversity indices, and abundances of dominant archaea. The depth of the color indicates the strength of each correlation. Red and blue colors indicate negative and positive correlation coefficients, respectively. The circle sizes reflect the significance of correlation coefficients. *, **, *** indicate significant correlation coefficients at *p* < 0.05, *p* < 0.01, and *p* < 0.001, respectively. WC, TC, TN, TP, DOC, and TFe denote peat water content, total carbon content, total nitrogen content, total phosphorus content, dissolved organic carbon, and total Fe content, respectively

To better demonstrate the interaction between the peat physicochemical properties and archaeal community, four representative indices characterizing *α*-diversity of the archaeal community were extracted (Fig. 7). Chao1 and observed species represent the richness of archaea. According to the obtained results, Chao1 was significantly and negatively correlated with pH and TP (*p* < 0.05), while the observed species showed a significant negative correlation with TP (*p* < 0.05). The Simpson diversity index of archaea exhibited strong positive correlations with TC and TN (*p* < 0.01), as well as with WC (*p* < 0.05). Whereas Pielou’s evenness index of the archaeal community was positively correlated with TN and WC (*p* < 0.05). Seven archaea orders were incorporated in the analysis as the sum of their relative abundances accounted for 94% of the top 20 archaea orders (Figs. 3 and 7). Among them, *Methanomicrobiales* and *Methanosarcinales* did not show any correlations with peat physicochemical parameters. *MBG–D/DHVEG–1* was negatively correlated with pH (*p* < 0.05) and positively correlated with WC (*p* < 0.05) and DOC (*p* < 0.01). *Methanobacteriales* showed a strong positive correlation with TFe (*p* < 0.01), while *Nitrosotaleales* and *Methanomethyliales* were negatively correlated with pH and TFe (*p* < 0.05). *Methanomassiliicoccales* exhibited significant positive correlations with TC (*p* < 0.05), C/N (*p* < 0.01), and pH (*p* < 0.001).

A redundancy analysis (RDA) was performed in this study to further investigate the relationships between the physicochemical properties of peat samples, dominant archaea, and peatlands. The first two axes explained 20.28% (adjusted) of the total variance, of which the first (RDA1) and second (RDA2) axes explained 12.70 (*p* = 0.001) and 7.58% (*p* = 0.009) of the total variance, respectively (Additional file 1: Fig. S1). The projections of the study areas with depths characterized by physicochemical properties and dominant archaea showed that H was contained in R. However, the 60–100 cm layer in R was comparatively separate, while H and R were both distinguished from T (Additional file 1: Fig. S1). Furthermore, several archaea were region-specific. For example, *Methanomassiliicoccales* was specific to the deep peat layer (60–100 cm) of R, showing qualitative correlations with C/N, pH, TC, WC, and TP, while *Methanobacteriales* and *Methanomicrobiales* were projected to the areas of H and R and were slightly correlated with WC, TFe, and C/N (Additional file 1: Fig. S1). *Nitrosotaleales* and *MBG–D/DHVEG–1* were observed in the T area and were correlated primarily with DOC, TP, TN, and TFe (Fig. S1), which is consistent with the archaeal distribution and correlation results (Figs. 3, 4, and 7). In addition, pH, TC, TN, and TFe were positively correlated with each other and negatively correlated with DOC and TP. Whereas TP was positively correlated with DOC and WC.

## Discussion

### Comparison of the three peatlands

The results obtained showed some similarities between the three peatlands. Indeed, all peatlands exhibited low pH values and high WC and TC (Table 1), corresponding to the suitable environmental conditions for peatland development [13, 19]. In addition, the results showed similar C/N values, demonstrating the homogeneity of biochemical processes in the three peatlands. The peat physicochemical properties showed minor influences on dominant archaea (20.28%, Additional file 1: Fig. S1), suggesting other factors affecting the archaeal community in these peatlands, including climate, plant species, and hydrological characteristics [75–78].

The pH values and the TFe contents in R were higher than those observed in the remaining peatlands (Table 1). This finding might be due to the dominance of water recharge by groundwater, transporting numerous mineral substances, including Fe, that contribute to the pool of basic cations that could be consumed by eluviation [15, 24], thereby maintaining comparatively high pH values [22]. In addition, due to the input of mineral substances, silicon might release bound phosphorus, thereby increasing the available phosphorus contents [79, 80]. Therefore, besides groundwater-derived nutrient elements, TP contents were also higher in R than those in the other peatlands. The DOC contents in R were comparatively low (Table 1), which might be due to the pH and hydrological conditions. Low pH values favor the bonding of acidic elements to organic matter [81], while the hydrological characteristics can change the DOC concentrations by affecting the input and output nutrients in peatlands [54]. Therefore, the minerotrophic fens could not accumulate DOC. Similarly, the increase in TC, TN, and C/N values with increasing peat depth suggested relatively strong microbial activity in deep peat layers in R (Table 1).

T is a *Sphagnum*-dominated ombrotrophic bog, exhibiting the lowest pH and TFe values and the highest DOC concentration (Table 1). The thickness of the peat layer in T can barely reach 1 m [27]. The results showed relatively low TC and TN contents in T (Table 1), which reflect its short development period. In addition, several peat physicochemical parameters showed significantly lower WC, DOC, C/N, TC, TN, and TP concentrations in the deeper peat layers than those observed in the surface peat layers (Table 1), suggesting the transition from peat to mineral soil and the weak biochemical activity in deeper peat layers. In addition, the unique karst landform in this area may also enhance groundwater recharge [26, 27], thereby promoting the release of mineral-bound phosphorus and, consequently, increasing phosphorus concentrations [79].

H is a typical poor fen characterized by negligible groundwater recharge [26]. Precipitation and surface runoff are the main water sources in H. In addition, the low infiltration rates often result in high groundwater levels above the peat surface [26, 51], explaining the highest WC in the three peatlands, as well as the high DOC and low TP contents (Table 1). H exhibited slight variations in the peat physicochemical parameters compared to the remaining peatlands (Table 1), suggesting stable biochemical processes and environmental changes in H.

### Archaeal community characteristics and dominant species with corresponding methanogenesis pathways

NMDS ordination revealed the gradient-related differences in the archaeal community in the three peatlands, showing slight and substantial changes in the archaeal community in the surface and deep peat layers among three peatlands, respectively (Fig. 2), which is relatively consistent with the RDA analysis of dominant archaea (Additional file 1: Fig. S1) and the results reported in previous studies [82–84]. The ability of NMDS to reflect the differences in the archaeal community in different peatlands was further confirmed by the stress (0.116) and fitness results (*R*^2^ = 0.986 for nonmetric fit, *R*^2^ = 0.929 for linear fit, Fig. 2) of the model, supporting the area-depth differentiation of the archaeal community in the three peatlands. Minor et al. (2019) also indicated differences in the plant communities and nutrient concentrations between different peatland types [42]. The results of the present study showed great variations in the archaeal community in T, which might be due to the large variation in the physicochemical properties in this peatland area (Table 1 and Fig. 2). The great impacts of peat physicochemical properties on the archaeal community structure (*α*-diversity) were further investigated in this study. According to the obtained results, TC, TN, TP, WC, and pH were the main factors affecting the archaeal community structure (Fig. 7), corresponding to the fact that peatland is an N/P-limited ecosystem [15, 24]. According to the results of the present study, peatland types can be accurately distinguished based on their physicochemical properties, plant communities, and microbial communities.

According to the relative abundance results of dominant orders in the archaeal community, *Methanosarcinales* and *Methanomicrobiales* were the main methanogenic orders in peatlands, showing similar distribution patterns (Figs. 3 and 4) and average relative abundances of 8%, which is consistent with the results reported in previous studies on peatlands in Yunnan Province, China, and Los Angeles, USA [83, 85]. The wide metabolic pathways and temperature growth ranges of these orders might be the main causes of their low correlations with environmental factors (Fig. 7) [33, 86, 87], suggesting their strong adaptability to the external environment. *Methanocellales* and *Methanofastidiosales* showed limited distributions in the three studied peatlands (Fig. 3). This finding might be because *Methanocellales* can only use H_2_/CO_2_ or formate to produce CH_4_ in a temperature range of 15–40 °C [87], while *Methanofastidiosales* are strictly methylotrophic methanogen rarely distributed in nature [33, 88]. *Methanobateriales*, which mainly use H_2_/CO_2_ or some simple methyl compounds to produce methane [86, 87], were more abundant in the upper peat layers in H and R (Figs. 3, 4, and Additional file 1: Fig. S1). However, *Methanobateriales* may exhibit a syntrophic relationship with certain bacteria, thereby coupling acetate oxidation and CH_4_ production processes [89]. Previous studies showed decreases in the abundance of bacteria with increasing peat depths as a result of decreases in the oxygen content [90, 91], and the syntrophic relationships between *Geobacter* and certain methanogens [92, 93], potentially explaining the high abundance of *Methanobateriales* in the upper peat layers and the positive correlation between *Methanobateriales* and TFe (Fig. 7). *Methanomassiliicoccales* and *Methanomethyliales* have similar metabolism pathways consuming methyl compounds [94, 95]. *Methanomassiliicoccales* were abundant in the 40–100 cm layer in R (Fig. 3 and Additional file 1: Fig. S1) and exhibited a strong positive correlation with pH (Fig. 7), suggesting that *Methanomassiliicoccales* are sensitive to acidic environments. Therefore, H and T might exhibit unsuitable conditions for *Methanomassiliicoccales* (Table 1). In contrast, *Methanomethyliales* were negatively correlated with pH (Fig. 7), suggesting good adaption of this order to acidic environments. Indeed, the obtained results revealed a higher relative abundance of *Methanomethyliales* in H compared to those in the remaining peatlands (Figs. 3 and 4).

The heatmap of the archaeal distribution revealed that *Methanobateriales*, *Methanomicrobiales* (*Methanofollis* and *Methanolinea*), *Methanocellales* (*Rice Cluster I* and *Methanocella*), and *Methanomethyliales* (*Candidatus Methanomethylicus*) were more abundant in H compared to other peatlands (Figs. 3 and 4), suggesting that hydrogenotrophic and methylotrophic were the major methanogenesis pathways in H [86, 87, 94, 95]. Whereas in R, *Methanomicrobiales*, *Methanomassiliicoccales* (*Methanomassiliicocus*), and *Methanosarcinales* (*Methanosarcina* and *Candidatus Methanoperedens*) were the dominant methanogens (Figs. 3, 4, and Additional file 1: Fig. S1), indicating that methyl compounds and acetate were the main substrates involved in the CH_4_ production process [87, 94]. The methanogenesis pathways in H and R were further investigated in this study by incubation experiments (Fig. 6a and c), indicating consistent results with the methanogenic composition (Figs. 3 and 4). Besides methanogens, *MBG–D/DHVEG–1* and *Nitrosotaleales* were also the dominant archaea in T (Figs. 3 and 6c). The relative abundance of these two archaea was about 78% of the methanogens in the 0–80 cm peat layer, which is considerably higher than that in the 80–100 cm peat layer in T (Fig. 3). The clustering results revealed a similar distribution of *Candidatus Bathychaeota RBG_16_48_13* to those of *Candidatus Methanomethylicus*, *Methanobrevibacter*, and *Rice Cluster I* (Fig. 4), suggesting their similarity in terms of their ecological functions and environmental adaptation. Some recent studies have highlighted similar methanogenesis pathways of *Bathyarchaeota* species to those of some methanogens (e.g., *Methanomassiliicoccales*) [33, 86, 96]. In addition, these species are capable of performing anaerobic mineralization of proteins and anaerobic CH_4_ oxidation [97, 98]. The RDA and correlation analysis revealed negative relationships between pH and *MBG–D/DHVEG–1* and positive relationships between DOC and *MBG–D/DHVEG–1* (Fig. 7 and Additional file 1: Fig. S1), suggesting that *MBG–D/DHVEG–1* may be adapted to acidic environments of bogs and sensitive to activated carbon components. *Nitrosotaleales* are adapted ammonia-oxidizing archaea to acidic environments [99, 100], which are involved in the nitrogen cycle and nitrous oxide emissions in peatlands [101, 102]. Abundant *Nitrosotaleales* were also found in the Dajiuhu peatland near the study site in T [102], indicating the wide distribution of this archaea in this area, possibly due to the particular karst landform [26, 27]. The results of the present study highlighted the presence of some other unique archaea in T, including *Aenigmarchaeales*, *Thermoplasmatales*, and *Deep-Sea Euryarchaeotic Group* (*DSEG*) (Figs. 3 and 4), suggesting the special local situation. Therefore, CH_4_ production in T might not be only controlled by traditional methanogens but also by other unique archaea through hydrogenotrophic methanogenesis, as revealed by the incubation experiment (Fig. 6b).

### Methanogenesis and its relationship with environmental factors and methanogens

The CH_4_ production potentials were significantly different between the three peatlands. The highest and lowest CH_4_ production potentials were observed in H and R, respectively (Fig. 5), which is consistent with the results reported in previous studies [43, 103, 104]. However, some studies conducted in boreal peatlands have indicated higher CH_4_ production in fens than in bogs [10, 43, 105]. In addition, other studies have revealed highly variable CH_4_ production potentials in peatlands due to their strong heterogeneity [43, 44, 106–108]. Ye et al. [109] found the highest CH_4_ production potential in the intermediate fen, which is similar to H. In contrast, Liu et al. [103] revealed extremely low and high CH_4_ production potentials in Zoige peatland (near R) and Sanjiang Plain in China, respectively, using incubation experiments, showing increasing trends in the CH_4_ production potential with increasing latitude. A previous study has also revealed high CH_4_ production potentials in bogs than those in other peatland types [110]. Therefore, the peatland types do not naturally determine the CH_4_ production potential.

The methanogenesis pathways in the different peatlands in our study, revealed by the archaeal community and incubation experiment results, are in line with the common rule (Figs. 3, 4, and 6) [23, 34, 111]. Generally, acetotrophic methanogenesis is dominant in minerotrophic fens, while methylotrophic methanogenesis occurs often in environments with high salt contents, such as marine and saline soda lakes where abundant methyl compound are available for methanogens [34, 62, 112, 113]. Previous studies have demonstrated that methanol is not the main substrate for CH_4_ production in peatlands [89, 114, 115]. In contrast, other studies have demonstrated that methanol could be the main substrate for methanogenesis in cold environments [63, 116], even in *Sphagnum*-dominated bogs [117], which is consistent with the results shown in the present study (Fig. 6a and c), suggesting the occurrence of methylotrophic methanogenesis in cold peatlands with high latitude. In addition, the CH_4_ production processes in H could be similar to that observed in T than R, even though there were some similar characteristics between H and R, such as methylotrophic methanogenesis. Some studies have shown that acetate can be formed from methanol through the fermentation process, thereby indirectly accelerating acetotrophic methanogenesis [62, 116]. However, our results were relatively inconsistent with these findings since the acetate addition in this study results in a minor enhancement effect on methane production, while the methanol addition significantly promoted methanogenesis in R (Fig. 6c).

The correlation analysis results highlighted a strong negative relationship between the pH values and CH_4_ production potential (Table 2). Whereas the covariance analysis revealed a significant interaction effect between pH and study area on CH_4_ production potential (Additional file 1: Tables S2 and S3), suggesting a variation in the pH effects among the three peatlands and is, therefore, a crucial and regional-specific factor controlling CH_4_ production potentials. This finding is, indeed, consistent with the results of previous studies investigating the pH impacts on the CH_4_ production process, showing a great effect of pH on CH_4_ production and the optimal pH for CH_4_ production higher than that in situ [58, 109, 110, 118].

DOC represents the activated carbon component, which is easily utilized by microbes, thus influencing their activity [119, 120]. In this study, DOC concentration was positively correlated with the CH_4_ production potential (Table 2). There are two mechanisms explaining the enhancement effect of DOC on CH_4_ production. First, some DOC-derived compounds can directly accelerate CH_4_ production [43, 121]; secondly, DOC inputs promote the reduction of Fe(III) [122], as well as the reduction of other electron acceptors, thereby mitigating the suppression effect caused by oxidized electron acceptors.

The results of the present study showed also a positive correlation between WC and CH_4_ production potentials (Table 2). The WC can indirectly reflect the hydrological conditions, aeration, and oxygen contents in peatlands that affect the microbial community [41, 123–125]. The high WC in H was due to the high groundwater level [26]. Besides the high DOC concentrations, it can be concluded that H provides good environmental conditions for CH_4_ production. However, oxidized materials can be transported to minerotrophic fens (e.g., R) through groundwater recharge, maintaining a comparatively stable redox potential in peatlands [126] and, consequently, inhibiting CH_4_ production. The low DOC and WC values could be the main reasons explaining the lower CH_4_ production potential in T than that in H (Table 1 and Fig. 5).

The influences of environmental factors on CH_4_ production are attributed mainly to methanogens. In this study, although *Methanomassiliicoccales* were the main methanogen that exhibited a negative correlation with CH_4_ production potentials (*R* =  − 0.716, Table 2), this finding is considered unreliable due to the extremely low relative abundance of *Methanomassiliicoccales* (< 1%, Fig. 3). Whereas other methanogens and the total relative abundance of methanogens were not associated with CH_4_ production potentials, showing the lack of correlation between the relative abundance of methanogens and CH_4_ production potentials, which is consistent with the results reported in previous studies [46, 104, 127, 128]. For this weak correlation, it can be comprehended as that the presence of methanogens were not active enough to produce expected amount of methane, corresponding with the evidence of *mcrA* gene/transcript which better demonstrates the methanogenesis activity of methanogens [23].

Besides the possible lack of substrates for CH_4_ metabolism in R, reflected by the low DOC contents (Table 1), the competition between different microbes could also be an important factor influencing methanogenesis. Vegetation in R consists mainly of grasses and sedges, which have substantially higher productivities than that of *Sphagnum* in the remaining peatlands [53, 129]. In addition, grasses and sedges can release photosynthetic carbon and increase oxygen concentrations in the rhizosphere [28, 83, 130, 131], thereby enhancing the activities of non-methanogen species [132] and, consequently, mitigating the activities of non-competitive methanogens [131, 133–135].

## Conclusions

This study demonstrated the similarities and differences in the archaeal community associated with CH_4_ production in three typical peatlands in China, namely H, T, and R. The *β*-diversity of the archaeal community determined using NMDS revealed the differences in the archaeal community between the three peatlands, particularly in the deep peat layers. The mean relative abundance of methanogens in peat layers ranged from 10 to 12%, regardless of peatlands. In addition, the results showed high abundances of *Methanosarcinales* and *Methanomicrobiales* in all peat samples, while *Methanobacteriales* were distributed mainly in the upper peat layer (0–40 cm). Besides methanogens, *MBG–D/DHVEG–1*, *Nitrosotaleales*, and some other orders of *Bathyarchaeota* exhibited high abundances, particularly in T, which is characterized by unique geological conditions, highlighting the diversity of archaea in peatlands. The distribution of methanogens was generally in line with the respective methanogenesis pathways of the three peatlands. On the other hand, pH, DOC, and WC of peatlands were the main factors controlling CH_4_ production potentials. However, no correlations were observed between methanogens and methane production potentials, suggesting the slight effect of relative abundances of methanogens on CH_4_ production in peatlands. The combined effect of these physicochemical factors resulted in the highest and lowest CH_4_ production potentials in H and R, respectively. In addition, the obtained results suggested that the peat physicochemical properties and archaeal community are important factors controlling methanogenesis in peatlands. Focusing on the ecological and biological aspects, this study provides a reference for investigating the relationship between the archaeal community and CH_4_ production process in different types of peatlands in China, highlighting the importance of the archaeal community and peat physicochemical properties for studies on methanogenesis in peatlands.

## Supplementary Information

**Additional file 1**: **Table S1**. Detailed information of the three study peatlands. **Table S2**. Significances of interactions between study area and each index. **Table S3**. An example of analysis of covariance used in Table S2. **Fig. S1**. Redundancy analysis of peat physicochemical properties with dominant archaea.

## Data Availability

Raw sequence data have been deposited in the Genome Sequence Archive (Genomics, Proteomics & Bioinformatics 2021) in the National Genomics Data Center (Nucleic Acids Res. 2022), China National Center for Bioinformation/Beijing Institute of Genomics, Chinese Academy of Sciences (GSA: CRA007847), publicly accessible at https://ngdc.cncb.ac.cn/gsa. Other data in this paper are available when requested.
