# Supplementary material for: Variations in the archaeal community and associated methanogenesis in peat profiles of three typical peatland types in China
Source: Environ Microbiome. 2023 Jun 6;18:48. doi: 10.1186/s40793-023-00503-y (PMC10246374; doi:10.1186/s40793-023-00503-y)
Supplement: Supplementary file 1 — Additional file 1: Table S1. Detailed information of the three study peatlands. Table S2. Significances of interactions between study area and each index. Table S3. An example of analysis of covariance used in Table S2. Fig. S1. Redundancy analysis of peat physicochemical properties with dominant archaea. [file 40793_2023_503_MOESM1_ESM.docx]

**Additional file for**

**Variations in the Archaeal community and associated methanogenesis in peat profiles of three typical peatland types in China**

**Xuhui Chen, Dan Xue*, Yue Wang, Qing Qiu, Lin Wu, Meng Wang, Jiawen Liu, Huai Chen***

Table S1. Detailed information of the three study peatlands.

| Peatland | Location | Climate type | MAT  (°C) | MAP  (mm) | Dominant  vegetation | Water  recharge | peatland  type |
| --- | --- | --- | --- | --- | --- | --- | --- |
| H | 126.51°E, 42.22°N | temperate continental climate | 6 | 750 | *Carex lasiocarpa*, *Phragmites australis*, *Eriophorum polystachion*, and *Sphagnum palustre* | surface runoff and precipitation, possiple minor gorund water | poor fen |
| T | 109.75°E, 29.96°N | subtropical monsoon climate | 8 | 1800 | *Sphagnum palustre, Juncus effusus*, *Miscanthus sinensis*, *Fargesia spathacea*, and *Corylus ferox* | precipitation, possible minor ground water | bog |
| R | 102.61°E, 33.06°N | continental alpine temperate monsoon climate | 2 | 650 | *Festuca nivina*, *Elymus nutans*, *Kobresia setchuanensis*, *Carex muliensis*, and *Kobresia tibetica* | ground water | fen |

H, Hani peatland; T, Taishanmiao peatland; R, Ruokeba peatland; MAT, mean annual temperature; MAP, mean annual precipitation.

Table S2. Significances of interactions between study area and each index. The numbers are *p*-values of interactions between study area and each index calculated by analysis of covariance with the formula “MPP ~ index * study area” where index represents each index in this table.

| – | WC | pH | DOC | *Methanomassiliicoccales* |
| --- | --- | --- | --- | --- |
| Study area | 0.852 | 0.005 | 0.955 | 0.088 |

WC and DOC are abbreviations of peat water content and dissolved organic carbon, respectively.

Table S3. An example of analysis of covariance (for pH) used in Table S2.

| – | Df | Sum of squares | Mean of squares | F-value | *p*-value |
| --- | --- | --- | --- | --- | --- |
| pH | 1 | 4665975 | 4665975 | 4357.23 | < 0.001 |
| Study area | 2 | 2313895 | 1156947 | 1080.39 | < 0.001 |
| pH: Study area | 2 | 104532 | 52266 | 48.81 | **0.00515** |
| Residuals | 3 | 3213 | 1071 | – | – |

Bold *p*-value is the reported value for pH in Table S2.





Figure S1. Redundancy analysis (RDA) of peat physicochemical properties with dominant archaea (top 20 orders). Different colors of points represent different peat depths shown in the legend. Peat samples from Hani, Taishanmiao, and Ruokeba are indicated by solid circles, triangles, and squares, respectively. Dashed ellipses are confidence intervals of samples from each peatland calculated by *t*-test (*α* = 0.05). Black arrows represent the projections of peat physicochemical properties (explanatory variables), and green arrows with red open circles represent the projections of archaea (response variables). The percentages shown in each axis are respective explanations for total variance corrected by adjusted *R^2^*. *P*-values were obtained with permutation tests (*n* = 999). Most of the archaea were crowded at the origin, thus not shown after selection. The abbreviations are consistent with those in Table S1, and MBG–D is the abbreviation of Marine Benthic Group D.
